# Comparisons of Muscle Quality and Muscle Growth Factor Between Sarcopenic and Non-Sarcopenic Older Women

**DOI:** 10.3390/ijerph17186581

**Published:** 2020-09-10

**Authors:** Myong-Won Seo, Sung-Woo Jung, Sung-Woo Kim, Hyun Chul Jung, Deog-Yoon Kim, Jong Kook Song

**Affiliations:** 1 Department of Taekwondo, College of Physical Education, Kyung Hee University, 1732 Deokyoungdaero, Giheung-gu, Yongin-si, Gyeonggi-do 17014, Korea; myongwonseo@khu.ac.kr; 2 Department of Physical Education, Graduate School, Kyung Hee University, 1732 Deokyoungdaero, Giheung-gu, Yongin-si, Gyeonggi-do 17014, Korea; jswoo@khu.ac.kr (S.-W.J.); kswrha@khu.ac.kr (S.-W.K.); 3 Department of Coaching, College of Physical Education, Kyung Hee University, 1732 Deokyoungdaero, Giheung-gu, Yongin-si, Gyeonggi-do 17014, Korea; jhc@khu.ac.kr; 4 Department of Nuclear Medicine, Kyung Hee University School of Medicine, 26, Kyungheedae-ro, Dongdaemun-gu, Seoul 02447, Korea; petct@daum.net; 5 Department of Sports & Science, Graduate School of Physical Education, Kyung Hee University, 1732 Deokyoungdaero, Giheung-gu, Yongin-si, Gyeonggi-do 17014, Korea

**Keywords:** sarcopenia, muscle growth regulators, muscle quality, functional performance

## Abstract

Sarcopenia, an age-related disease, is one of the important health problems in the elderly and the prevalence of sarcopenia is rapidly increased among the Korean population. This study examined the muscle quality and muscle growth factors of elderly women to identify the potential diagnostic tool for sarcopenia. One hundred and thirty-six elderly women, aged over 65 years old, initially enrolled, but only 59 participants who met the criteria (sarcopenic group, *n* = 27; non-sarcopenic group, *n* = 32) completed the study. Muscle quality assessment included thigh cross-sectional computed tomography scan and maximal isometric muscle strength. Muscle growth factors such as GDF-15, myostatin, activin A, and follistatin were analyzed, and a battery of Senior Fitness Test was used to examine functional fitness. The statistical significance level was set at 0.05. Elderly women with sarcopenia had a lower thigh muscle volume (−20.1%), and a higher thigh intermuscular adipose tissue (15.8%) than those of the non-sarcopenic group (*p* < 0.05). However, no significant differences in muscle growth factors were observed between the groups. Muscle quality variables including maximal voluntary isometric contraction (OR: 0.968, *p* < 0.001), relative maximal voluntary isometric contraction (OR: 0.989, *p* < 0.05), thigh muscle volume (OR: 0.836, *p* < 0.001), and thigh intermuscular adipose tissue (OR: 1.138, *p* < 0.05) were associated with a risk of sarcopenia. Our findings suggest that the sarcopenic group exhibits a poor thigh muscle quality in comparison with the non-sarcopenic group. Muscle quality assessment can be utilized for sarcopenia identification, but our study remains inconclusive for the causality of muscle growth factors in sarcopenia.

## 1. Introduction

Sarcopenia is an age-related disease described by a progressive loss of muscle mass, function, and performance [1]. It has become a major public health problem because the prevalence of sarcopenia is rapidly increasing all over the world. A systematic review and meta-analysis reported that the overall estimated prevalence of sarcopenia is 10% (Men, 95% CI: 8–12%; Women, 95% CI: 8–13%) in older adults [2], and this rate increases by more than 50% for those aged 80 years old or above [3]. This unfavorable trend has been associated with the risk of geriatric syndromes, poor quality of life, functional impairment, disability, morbidity, and mortality. 

It has been well recognized that age-related loss of muscle mass can be attributed to the complex interactions among factors including alterations of the neuromuscular junction, endocrine system, growth factor, muscle protein turnover, and behavior-related and disease-related factors. Accordingly, sarcopenia cannot be assessed by the use of a single biomarker due to its multifactorial pathogenesis. Protein carboxylation and advanced glycation end products (AGEs) are independently associated with low grip strength in women [4,5]. High levels of inflammation markers, such as C-reactive protein, TNF-α, and IL-6, were associated with decreased muscle mass and strength [6,7,8], while low serum albumin concentrations, testosterone, and 25-hydroxyvitamin D levels were identified as risk factors of sarcopenia and/or myopathy [9,10,11,12]. Recently, studies have introduced muscle-related biomarkers, including growth differentiation factor-15 (GDF-15), GDF-8 (myostatin), activin A, and follistatin that could be used as identifiable markers of sarcopenia [13,14,15], but a lack of studies may limit the confirmation of predictable biomarkers. Therefore, careful approaches to determine as biomarkers for sarcopenia are needed and, importantly, more attention should be paid to the direct indicators in diagnostic criteria for sarcopenia despite its significant association with muscle metabolism and function. 

Muscle quality, which is defined as muscle strength or power per unit of muscle mass, is a key determinant of muscle function in later life. It is mediated by many factors, such as type II fibers, fat infiltration, and neurological derangements. The European Working Group on Sarcopenia in Older People (EWGSOP) proposed the necessity to include muscle quality assessments for a diagnostic tool in sarcopenia [16], because muscle quality data could be used effectively to identify older adults who are at risk of impaired mobility. Although several methods, such as dual-energy X-ray absorptiometry (DXEA) and bioelectrical impedance analysis (BIA), have been utilized for the assessment of muscle quality, computed tomography represents the gold standard and the most accurate imaging method to provide an exact measurement of total cross-sectional area, muscle density, and subcutaneous and intermuscular adipose tissue. Particularly, age-related muscle loss is predominantly detected at the specific body site (i.e., thigh muscle) and the changes of thigh muscle quality have been associated with impaired mobility and other chronic diseases [17,18]. 

Since sarcopenia has received medical attention in public health, numerous inflammation markers have been examined to identify its risk factors. However, there is limited information about the muscle-specific biomarkers and whether these markers can be identified as a diagnostic tool of sarcopenia. Additionally, the assessments of thigh muscle quality can provide insightful information to detect pre-sarcopenia in older adults. Therefore, the purposes of this study were to investigate differences in muscle quality and muscle growth factors (i.e., GDF-15, myostatin, activin A, and follistatin) between sarcopenia and non-sarcopenia in elderly women and identify the potential diagnostic tool that develops sarcopenia in this population.

## 2. Materials and Methods

### 2.1. Participants

**Figure 1:**
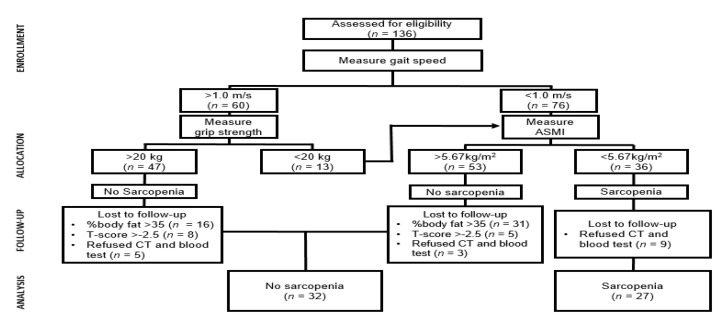
Flow chart of the study.

Participants were recruited via advertisement at senior citizen centers located in Suwon-city, Korea. Initially, one hundred and thirty-six older women aged 65–88 years old enrolled voluntarily in the study. To select eligible participants, we applied the adjusted diagnostic algorithm based on EWGSOP and IWGS (International Working Group on Sarcopenia) to define sarcopenia in older adults. The inclusion criteria for the sarcopenic group were (1) gait speed < 1.0 m·s^−1^ and appendicular skeletal muscle index (ASMI) 5.67 < kg∙m^−2^, or (2) gait speed > 1.0 m·s^−1^ grip strength < 20 kg and ASMI < 5.67 kg∙m ^−2^. The non-sarcopenic group included individuals who had (1) non-sarcopenic, (2) percent body fat < 35%, and (3) a lumbar or femur bone mineral density T-score < −2.5. The flow chart of the study is presented in Figure 1. Participants who did not meet these criteria were excluded from the study. After the initial screening, seventy-six participants were identified as meeting the criteria for this study. Seventeen participants dropped out due to personal reasons. Therefore, fifty-nine old women participated in this study (sarcopenic group: *n* = 27, age: 71.6 ± 4.11; non-sarcopenic group: *n* = 32; age: 71.4 ± 4.88). All participants were informed of the purpose of the study as well as its procedure, benefits, and possible risks by the principal investigator, and they completed an AHA/ACSM Health/Fitness Facility Pre-Participation Screening Questionnaire to screen for potential risk factors for old adults enrolling in this study. This study was approved by the Institutional Review Board of Kyung Hee University (KHSIRB-18-021), and written informed consent was obtained from all participants.

### 2.2. Anthropometric Measurements

Participants’ height and body weight were measured using a stadiometer (T.K.K. Takei Scientific Ins Co., Tokyo, Japan) and a digital scale (150A, CAS, Seoul, Korea), respectively. Body mass index (BMI) was calculated as weight in kilograms divided by the square of the height (kg·m^−2^). Waist circumference (WC) was measured at the midpoint between the lower rib margin and the iliac crest, and hip circumference (HC) was taken at the widest portion of the hip. Waist-to-hip ratio (WHR) was calculated as WC divided by HC. The anthropometric characteristics of height, weight, WC, and HC were measured with minimal light indoor clothing and without shoes.

### 2.3. Body Composition and Bone Mineral Density

Body composition (i.e., body fat percentage, fat mass, and lean body mass) as well as bone mineral density (BMD) of the whole body, lumbar spine (L1–L4), and proximal femur of the left leg were measured with a dual X-ray absorptiometry (DXA; QDR-4500W, Hologic, Marlborough, MA, USA). A DXA scan was performed barefoot wearing light cloth and no metal objects on the body. ASMI was calculated from the sum of lean mass for arms and leg divided by the square of the height. The coefficient of variance of DXA was approximately 1.5% or less, as indicated by the manufacturer. The DXA scan was performed by an experienced technician in our laboratory, and the intraclass correlation coefficient (ICCs) of DXA measurement was 0.99.

### 2.4. Functional Fitness Test

The senior fitness test (SFT), including chair stand, arm curl, 2-min step test, chair-sit-and-reach, back scratch, and 2.4 m up-and-go tests, were used to assess the functional fitness of older adults. The SFT battery was developed and validated by Rikli and Jones to predict the risk of impaired mobility and physical independence [19]. Additionally, grip strength (T.K.K. 5001, Takei, Japan) and gait speed (4-m, m·s^−1^) were measured to determine the sarcopenia. The ICCs of functional fitness tests ranged from 0.80 to 0.98.

### 2.5. Muscle Quality Assessment

Isometric muscle strength was measured with an isokinetic dynamometer (Cybex Humac Norm Model 770, Computer Sports Medicine Inc., Stoughton, MA, USA). The participants were seated on the dynamometer with their dominant knee flexed at about 60°, with their chest, hip, and leg tightly fixed with a belt to minimize the engagement of other joints. The participants performed three trials of maximal isometric knee extensions for 3 s with a 30 s interval between the trials. The reliability of the isokinetic dynamometer was 0.97 for the isometric peak torque of the knee extensor. The computed tomography (CT) estimated the cross-sectional area of total thigh volume (TTV), thigh fat volume (TFV), thigh muscle volume (TMV), thigh subcutaneous fat volume (TSFV), and intramuscular fat area (thigh intermuscular adipose tissue; IMAT). A bilateral CT scan of the thigh was acquired, and participants were placed in the supine position in Brivo CT385 (Brivo CT385, GE Healthcare, Chicago, IL, USA). The midpoint of the thigh was measured in the horizontal plane between the intercondyloid and the medial edge of the greater trochanter of the bilateral leg. Bilateral thigh muscle and adipose tissue cross-sectional areas were based on radiographic attenuation (Houndsfield unit, HU) with a threshold range of 0 to +100 HU used for muscle area and a range of −190 to −30 HU for adipose tissue. The ICC of CT scans was 0.99.

### 2.6. Biochemical Markers

Venous blood samples were collected in a laboratory between 8:00–9:00 a.m. after at least 12 h of overnight fasting. Participants were instructed to refrain from moderate or strenuous physical activity for 48 h before blood collection. Venous serum samples (5 mL) were taken from the antecubital vein and collected into a vacuum tube by a medical laboratory technologist. Blood samples were centrifuged at 3000 rpm for 10 min after 30 min of blood clotting at room temperature, and the separated serum samples were stored at −80 °C. All blood variables were analyzed by an automatic ELISA (enzyme-linked immunosorbent assay) microplate reader (VERSA Max Molecular Devices, Inc., Sunnyvale, CA, USA) with a GDF-15 kit (Human GDF-15 Immunoassay, R&D, USA), a GDF-8 kit (Myostatin Immunoassay ELISA Kit, R&D, Minneapolis, MN, USA), an activin A kit (Human Activin A Immunoassay ELISA Kit, R&D, Minneapolis, MN, USA), and a follistatin kit (Human Follistatin Immunoassay, R&D, Minneapolis, MN, USA). Biochemical analyses were performed at the standard research lab (Green Cross Lab Cell, certified by the Korea laboratory accreditation scheme, Korea). The inter- and intraclass coefficient of variance were 4.7% and 1.8% for GDF-15, 3.1% and 1.8% for GDF-8, 4.7% and 4.2% for activin A, and 5.7% and 1.7% for follistatin, respectively.

### 2.7. Questionnaire

Physical activity was assessed using the Korean version of the International Physical Activity Questionnaire (IPAQ, long version). The score of IPAQ was assessed for the duration and frequency of walking and moderate-to-vigorous physical activity. The nutritional intake was examined with three-day dietary records that were recorded twice on weekdays and once at the weekend. The data were analyzed by the computer-aided nutritional analysis software program (CAN-PRO 4.0, Korean Nutrition Society, Korea).

### 2.8. Statistical Analysis

All statistical analyses were performed by the SPSS software program (version 25, SPSS Inc., Chicago, IL, USA). Descriptive statistics were expressed as mean, standard deviation (SD), and 95% confidence interval (95% CI). An independent sample *t-*test was applied to compare dependent variables between sarcopenic and non-sarcopenic groups. The effect size (ES) was calculated using Cohen’s d equation (small effect ≥ 0.2, medium effect ≥ 0.5, large effect ≥ 0.8) [20]. The diagnostic tool for outcome was analyzed by comparing sarcopenic and non-sarcopenic groups through univariate logistic linear regression, which is expressed as an odds ratio (OR) with corresponding 95% CI. The significant level was set at 0.05.

## 3. Results

Table 1 shows the results of anthropometric characteristics, body composition, bone mineral density, physical activity, and nutritional intake between sarcopenic and non-sarcopenic groups. The sarcopenic group represented significantly lower BMI (*p* < 0.05, ES = 0.54, 95% CI 0.07, 2.23), lean body mass (*p* < 0.001, ES = 1.38, 95% CI 2.60, 5.75), ASMI (*p* < 0.001, ES = 1.58, 95% CI 1.27, 2.62), whole-body BMD (*p* < 0.001, ES = 1.27, 95% CI 0.03, 0.10), lumbar BMD (*p* < 0.001, ES = 1.27, 95% CI 0.03, 0.15), whole-body BMD T-score (*p* < 0.001, ES = 0.96, 95% CI 0.32, 1.11), lumbar BMD T-score (*p* < 0.001, ES = 0.96, 95% CI 0.32, 1.11), total dietary intake (*p* < 0.01, ES = 0.71, 95% CI 51.44, 327.07), lipid (*p* < 0.05, ES = 0.70, 95% CI 2.21, 16.22), protein (*p* < 0.01, ES = 0.71, 95% CI 2.29, 14.60), and a higher body fat percentage (*p* < 0.001, ES = 1.23, 95% CI −6.56, −2.67) than the non-sarcopenic group. However, there were no significant differences for age, height, weight, WC, HC, WHR, fat mass, femur BMD T-score, physical activity level, and carbohydrates between groups.

Table 2 illustrated the results of functional fitness tests. Functional fitness such as 30-s arm curl (*p* < 0.05, ES = 0.53, 95% CI 0.04, 3.44), grip strength (*p* < 0.001, ES = 1.16, 95% CI 2.11, 5.65), and gait speed (*p* < 0.01, ES = 0.84, 95% CI 0.04, 0.18) were significantly lower in sarcopenic than non-sarcopenic groups. However, no significant differences were observed in other variables between groups.

There were significant differences in maximal voluntary isometric contraction (MVIC; *p* < 0.01, ES = 0.88, 95% CI 10.01, 39.25), relative maximal voluntary isometric contraction (RMVIC; *p* < 0.05, ES = 0.60, 95% CI 4.48, 64.44), TMV (*p* < 0.001, ES = 1.44, 95% CI 9.65, 20.90), and IMAT (*p* < 0.05, ES = 0.57, 95% CI −5.19, −0.29) between sarcopenic and non-sarcopenic groups. However, no significant group differences in THV, TFV, and TSFV were observed. In this study, circulating levels of GDF-15, GDF-8, activin A, and follistatin were observed, and none of the biomarkers were significantly different between the sarcopenic and non-sarcopenic groups. The results of muscle quality and biomarkers are described in Table 3.

Logistic linear regression was used to identify potential risk factors of sarcopenia in elderly women (Table 4). A univariate analysis resulted in muscle quality variables being significantly associated with sarcopenia: maximal voluntary isometric contraction (OR: 0.968, *p* < 0.001, 95% CI 0.947, 0.990), relative maximal voluntary isometric contraction (OR: 0.989, *p* < 0.05, 95% CI 0.979, 0.999), thigh muscle volume (OR: 0.836, *p* < 0.001, 95% CI 0.756, 0.923), and thigh intermuscular adipose tissue (OR: 1.138, *p* < 0.05, 95% CI 1.008, 1.284).

## 4. Discussion

Sarcopenia is highly prevalent, and the risk of sarcopenic-related comorbidities has rapidly increased among the Korean population. This study examined the physiological and physical characteristics of sarcopenic elderly women by a comparison between sarcopenic and non-sarcopenic groups. We focused on muscle quality and muscle growth factors to identify the diagnostic tool of sarcopenia in elderly women. The major findings of the study indicated that muscle quality including MVIC, TMV, and IMAT was significantly lower in the sarcopenic group than the non-sarcopenic group, but muscle growth factors were not different between the groups. The sarcopenic group was associated with poor muscle quality, such as lower in MVIC, RMVIC, and TMV as well as higher in IMAT when compared to the non-sarcopenic group. 

Since the sarcopenia has received considerable research attention, various diagnostic criteria have been introduced [16]. In the present study, thirty-six participants (26.5%) out of one hundred and thirty six elderly female volunteers were categorized into the sarcopenic group. We applied the adjusted cut-off values that have been used in EWGSOP and IWGS. However, this criterion is relatively more liberal than any other criterion methods that have been introduced by EWGSOP, the Asian Working Group for Sarcopenia (AWGS), the Foundation for the National Institutes of Health (FNIH), and IWGS. There were also different numbers of individuals who were classified as sarcopenic based on different criterion methods (EWGSOP, *n* = 10, 7.4%; AWGS, *n* = 8, 5.9%; FNIH, n=6, 4.4%; IWGS, *n* = 42, 31.1%) in the present study. Although the use of those criterion methods to diagnose for pre-sarcopenia and/or sarcopenia are common in society, numerous challenges exist that may limit their application in research and clinical settings because utilizing different algorisms, measurement tools (i.e., DXA, BIA), upn different populations (i.e., ethnicity) can influence the result of the prevalence rate within the group or country. For instance, a cohort study reported that the prevalence of sarcopenia showed a wide range from 4.7 to 16.2% (EWGSOP: 16.2%, AWGS: 8.0%, FNIH: 4.7%) among Korean older women [21]. Therefore, the optimal definition in sarcopenia needs to be continuously developed for each ethnicity, and some effort would be required to develop a standardized diagnosis system for pre-sarcopenia and/or sarcopenia. 

The present study found that BMI, lean body mass, and ASMI were significantly lower while body fat percentage was higher in elderly women with sarcopenia. Our findings suggest that the assessment of anthropometric indexes such as BMI needs special consideration for the older population. Sarcopenia in older adults presents a lower BMI compared to obesity in older adults despite a higher body fat percentage [22], and this incorrect information could induce the misleading of evaluation and treatment, especially sarcopenic obesity in older adults. Moreover, BMI cannot provide accurate body composition data such as fat and muscle. The FNIH Sarcopenia Project used that criteria using BMI-adjusted appendicular skeletal muscle mass (ASM/BMI) for clinical diagnosis of sarcopenia [23]. Therefore, BMI values should be used for skeletal muscle mass index, adjusted for accurate sarcopenia diagnosis in the older population. Verlaan et al. demonstrated that older adults with sarcopenia had a significantly lower ASMI (kg/m^2^) compared to non-sarcopenic older adults [24]. Another study reported that sarcopenic older adults were lower in lean body mass than the non-sarcopenic group without osteoporosis [25]. Different results between studies are particularly due to the classification and the adjusted ASMI score. Interestingly, total body fat mass was not different between groups in this study. A previous study supports our result that body fat percentage, rather than fat mass, is a more important predictor of mortality [26], because the decreased muscle mass by age or physical inactivity contributes more to functional immobility than fat accumulation. Additionally, the loss of muscle mass directly affects bone health, where our result confirms that the whole body and the lumbar BMD were lower in the sarcopenic group. A previous study reported that the sarcopenic group was significantly lower in lumbar BMD than the non-sarcopenic group, and adjusted odds ratios in potential risk factor were 1.88 (95% CI: 1.22, 2.89) and 1.72 (95% CI: 1.09, 2.72) in lumbar BMD (model 1: age and BMI; model 2: age, BMI, exercise time, osteoporosis agents use, vitamin-mineral supplement use, menopause, and hormone replacement therapy) [27]. Moreover, Lima et al. demonstrated that the prevalence of osteoporosis in older women was 15.8% for non-sarcopenia, 19.2% for pre-sarcopenia, 35.3% for sarcopenia, and 46.2% for severe sarcopenia, respectively [28]. Especially, our findings revealed that approximately 87% of the sarcopenia group had osteoporosis (26.6%) or osteopenia (60%). 

Functional fitness is a critical component to estimate the abilities of independent living in older adults, and it is associated with the risk of mobility. This study found that the sarcopenic group showed significantly lower in 30-s arm curl, grip strength, and gait speed than the non-sarcopenic group. Tang et al. and Gray et al. reported that older adults with sarcopenia had lower grip strength and gait speed than older adults without sarcopenia [29,30]. However, there were no significant differences in 30-s chair stand, chair sit-and-reach, back scratch, 8-foot up-and-go, and 2-min step tests between groups. Future studies with strict enrollment criteria and a large sample size are required to clarify the contradiction of relations between sarcopenia and functional fitness in older adults.

Alongside weak functional fitness level, poor muscle quality is often identified in older adults with sarcopenia. Several factors such as neurological derangements, fat infiltration, and proportion of type II muscle fiber have been introduced as the cause of poor muscle quality [31,32]. Particularly, the assessment of site-specific muscle quality such as thigh muscle is crucial to determine functional mobility as well as healthy life expectancy. The present study revealed that isometric muscle strength was significantly lower in the sarcopenic group than in the non-sarcopenic group. It is expected that a decrease in muscle strength is greatly affected by the loss of muscle mass and the changes in muscle quality. Our sarcopenic group also demonstrated a lower muscle quality including TMV and IMAT compared to the non-sarcopenic group. While various techniques have been used to assess muscle mass, few have been incorporated into routine use in older adults. A computed tomography (CT) is widely used as the “Gold Standard” for diagnosis. However, this tool was not commonly used in primary diagnosis and treatment because of high costs and a limited availability of equipment. Nevertheless, with ever-increasing requirments to assess the architecture and tissue composition of sacopenia and to identify it in preliminary diagnosis, high-resolution computed tomography is predicted to be generally utilized in future clinical practice and in research areas. Especially, evidence has shown that thigh muscle volume is positively associated with muscle function. Tsukaski et al. suggested that the cross-sectional muscle area of the mid-thigh, assessed by CT or DXA, is an important indicator of mobility capacity, which is considered to be a practical approach for sarcopenia diagnosis [33]. Notably, thigh muscle attenuation was inversely associated with the incident hip fractures, and after adjusting for compound covariates, the hazard ratio was 1.51 (95% CI 1.13, 2.03, model; age, race, gender, clinic, height, BMI, percentage of fat, self-reported health, chronic disease index, and physical activity) [34]. It is assumed that the accumulation of intermuscular fat is predisposed to subcutaneous fat accumulation, and fat infiltration can play a key role in the pathophysiological process of various diseases, such as muscle atrophy, cardiovascular diseases, and metabolic syndrome. Visser et al. reported that muscle fat infiltration status is associated with mobility limitation in both males and females, and adjusted hazard ratios in risk factors were ranged from 1.98 (female; 95% CI, 1.43–2.76) to 2.16 (male; 95% CI, 1.48–3.14) in mobility impairments (model: age, race, study site, body height, and total body fat mass) [10]. Moreover, future studies are needed to develop the sarcopenia diagnosis of the mid-thigh muscle area and should assist in the exact definition of the clinical classification in sarcopenia and older adults.

In the present study, muscle growth factors were not significantly different between the sarcopenic and non-sarcopenic groups, and none of the variables were associated with the diagnostic tool of sarcopenia. GDF-15 induces atrophy of C2C12 myotubes and is associated with acute muscle atrophy in heart failure and cachexia [35]. Hofmann et al. and Framz et al. reported that there was no significant difference in GDF-15 between sarcopenic and non-sarcopenic groups [36,37]. However, a cohort study by Rothenbacher et al. demonstrated that GDF-15 was positively associated with inflammation markers and was negatively related to lipid profiles, gait speed, and grip strength independent of age and gender [38]. These inconsistent outcomes between studies may be influenced by the disease severity in patients and different expressions between protein and mRNA. Myostatin belongs to the transforming growth factor-β (TGF-β) family, which is mainly secreted from skeletal muscle fibers. Previous studies had shown that no significant difference was observed in the myostatin levels between young and sarcopenic older adult men [39], and sarcopenic and non-sarcopenic older adults [40]. However, the secretion of myostatin increased 2.9 fold in severely obese women [41], and the activation of myostatin was suppressed by the AMPK and lipid metabolism in the peripheral tissues [42]. Moreover, myostatin-deficient mice were associated with fat accumulation [43]. It is assumed that fat metabolism may play an important role in the regulation of myostatin. Our study confirmed that myostatin may not be the primary cause of sarcopenia. Activin A is one of a TGF-β superfamily member that is primarily expressed in the reproductive tissues. Our results are inconsistent with the findings of Hofmann et al., where the sarcopenic group had higher activin A than the non-sarcopenic group. Moreover, serum levels of activin A was higher in cachectic patients than in non-cachectic patients [44]. Activin A is associated with many complex diseases including cancer, sarcopenia, metabolic syndrome, and cardiovascular disease [45]. Follistatin is a strong antagonist of the TGF-β superfamily induced by muscle atrophy. A recent cohort study with a population of 463 (mean age: 69.1) has confirmed that there was no difference in follistatin levels between the sarcopenic and non-saropenic groups [46]. Another study also reported that serum levels of follistatin were not different among young males, older men with mild sarcopenia, and older men with severe sarcopenia [40]. However, a negative correlation between follistatin and gait speed was observed in older adults and patients with chronic kidney disease [47,48]. These conflicting results might be affected by the fact that TGF-β member (e.g., GDF-8, GDF-15, and Activin A) status may be accompanied by an increase in follistatin [47]. However, the present study was unable to address the question of the causality of muscle growth factors in sarcopenia. In fact, because of the complicated underlying pathophysiology responsible for the muscle metabolism of sarcopenia, it is doubtful to confirm an individual’s status in older adults with and without sarcopenia using muscle growth factors [49]. Nevertheless, future studies should be performed to identify potential biomarkers for sarcopenia diagnosis.

## 5. Conclusions

This comparative study examined muscle quality and muscle growth factors to identify the risk of sarcopenia. Our findings indicate that the sarcopenic group exhibits lower site-specific thigh muscle quality including isometric muscle strength, total muscle volume, and intramuscular adipose tissue in comparison with the non-sarcopenic group. Muscle quality assessment can be utilized to identify the risk of sarcopenia, but our study remains inconclusive for the causality of muscle growth factors in sarcopenia.

## Figures and Tables

**Table 1:** Comparison of anthropometric, body composition, bone mineral density, physical activity, and nutritional intake between sarcopenic and non-sarcopenic groups.

Variable		Sarcopenic (*n* = 27)	Non-Sarcopenic (*n* = 32)	*t*-Value	Cohen’s *d*	95% CI	
						Lower	Upper
Anthropo metric	Age (yr)	71.4 ± 4.88	71.6 ± 4.11	−0.15	0.04	−2.16	2.52
	Height (cm)	153.1 ± 5.23	153.2 ± 5.89	−0.08	0.02	−2.82	3.04
	Weight (kg)	52.7 ± 4.88	55.5 ± 5.77	−1.97	0.52	−0.05	5.58
	BMI (kg·m^−2^)	22.5 ± 1.80	23.6 ± 2.28	−2.14 *	0.54	0.07	2.23
	WC (cm)	76.3 ± 4.82	78.8 ± 6.87	−1.58	0.42	−0.67	5.63
	HC (cm)	90.2 ± 3.92	91.6 ± 4.20	−1.36	0.34	−0.69	3.57
	WHR (ratios)	0.85 ± 0.04	0.86 ± 0.06	0.98	0.20	−0.01	0.04
Body compositon	Fat mass (kg)	18.1 ± 3.28	16.6 ± 3.01	1.91	0.48	−3.21	0.07
	% Body fat (%)	34.9 ± 3.92	30.3 ± 3.55	4.74 ***	1.23	−6.56	−2.67
	LBM (kg)	32.0 ± 2.43	36.1 ± 3.42	−5.30 ***	1.38	2.60	5.75
	ASM (kg)	12.6 ± 1.02	14.6 ± 1.47	−5.82 ***	1.58	1.27	2.62
Bone mineral density	WBMD(g·cm^−2^)	0.93 ± 0.06	1.00 ± 0.07	−3.68 ***	1.27	0.03	0.10
	WBMD Tscore	−1.92 ± 0.70	−1.20 ± 0.79	−3.64 ***	0.96	0.32	1.11
	LBMD (g·cm^−2^)	0.80 ± 0.10	0.88 ± 0.12	−3.01 ***	0.79	0.03	0.15
	LBMD T-score	−1.82 ± 0.87	−1.05 ± 1.01	−3.10 ***	0.82	0.27	1.27
	FBMD (g·cm^−2^)	0.70 ± 0.10	0.74 ± 0.08	−1.69	0.44	−0.01	0.87
	FBMD T-score	−1.30 ± 0.88	−0.96 ± 0.69	−1.66	0.43	−0.07	0.75
Physical activity	PA level (MET-min·week^−1^)	1690.2 ± 1128.69	1784.4 ± 1553.49	−0.262	0.07	−625.86	814.30
Nutritional intake	TDI (kcal·day^−1^)	1222.9 ± 301.99	1412.1 ± 225.94	−2.750 **	0.71	51.44	327.07
	Carbohydrate (g)	198.0 ± 49.61	214.3 ± 38.29	−1.420	0.37	−6.67	39.19
	Lipid (g)	29.0 ± 11.10	38.2 ± 15.04	−2.633 *	0.70	2.21	16.22
	Protein (g)	47.9 ± 13.46	56.4 ± 10.14	−2.746 **	0.71	2.29	14.60

Values are expressed as mean ± SD. Significant difference between sarcopenic and non-sarcopenic groups:, * *p* < 0.05, ** *p* < 0.01, and *** *p* < 0.001. BMI: body mass index, WC: waist circumference, HC: hip circumference, WHR: waist to hip ratios, LBM: Lean body mass, ASM: appendicular skeletal muscle, WBMD: whole body bone mineral density, LBMD: lumbar bone mineral density, FBMD: femur bone mineral density, PA: physical activity, TDI: total dietary intake, 95% CI: 95% confidence interval.

**Table 2:** Comparison of functional fitness between sarcopenic and non-sarcopenic groups.

Variable	Sarcopenic (*n* = 27)	Non-sarcopenic (*n* = 32)	*t*-Value	Cohen’s *d*	95% CI	
					Lower	Upper
30-s chair stand (n)	14.6 ± 4.15	14.8 ± 2.79	−0.21	0.06	−1.70	2.09
30-s arm curl (n)	15.9 ± 3.66	17.6 ± 2.64	−2.06 *	0.53	0.043	3.44
Chair sit-and-reach (cm)	15.5 ± 11.19	15.6 ± 9.55	−0.05	0.00	−5.28	5.53
Back scratch (cm)	−2.4 ± 6.70	−4.1 ± 7.56	0.90	0.24	−5.44	2.07
8-foot up-and-go (s)	5.8 ± 0.57	5.7 ± 0.81	0.23	0.14	−0.41	0.33
2-min step test (n)	89.9 ± 11.50	92.3 ± 15.61	−0.67	0.18	−4.85	9.69
Grip strength (kg)	20.4 ± 3.27	24.3 ± 3.47	−4.39 ***	1.16	2.11	5.65
Gait speed (m·s^−1^)	0.96 ± 0.12	1.07 ± 0.14	−3.22 **	0.84	0.04	0.18

Values are expressed as mean ± SD. Significant difference between sarcopenic and non-sarcopenic groups: * *p* < 0.05, ** *p* < 0.01, and *** *p* < 0.001; 95% CI: 95% confidence interval.

**Table 3:** Comparison of muscle quality and biomarkers between sarcopenic and non-sarcopenic groups.

	Variable	Sarcopenic (*n* = 27)	Non-Sarcopenic (*n* = 32)	*t*-Value	Cohen’s d	95% CI	
						Lower	Upper
Muscle quality	MVIC (N·m)	111.0 ± 28.59	135.6 ± 27.37	3.374 **	0.88	10.01	39.25
	RMVIC(N·m·kg^−1^)	212.5 ± 57.56	246.9 ± 57.07	2.301 *	0.60	4.48	64.44
	TTV (cm^2^)	143.7 ± 18.31	153.1 ± 20.40	1.844	0.48	−0.8	19.58
	TFV (cm^2^)	63.2 ± 13.67	56.3 ± 13.51	−1.932	0.51	−13.96	0.25
	TMV (cm^2^)	75.5 ± 8.35	90.7 ± 12.42	5.614 ***	1.44	9.65	20.90
	TSFV (cm^2^)	46.2 ± 13.29	41.9 ± 12.35	−1.262	0.34	−10.91	2.47
	IMAT (cm^2^)	17.1 ± 5.37	14.4 ± 4.02	−2.240 *	0.57	−5.19	−0.29
Muscle growth factor	GDF-15 (pg·mL^−1^)	835.67 ± 335.03	741.54 ± 349.00	1.051	0.28	−273.45	85.20
	GDF-8 (pg·mL^−1^)	2006.99 ± 729.75	2064.24 ± 869.18	−0.271	0.07	−365.85	480.36
	Activin A (pg·mL^−1^)	380.17 ± 90.23	351.26 ± 97.45	1.174	0.31	−78.21	20.40
	Follistatin (pg·mL^−1^)	2092.43 ± 562.30	2203.62 ± 588.78	−0.738	0.19	−190.67	413.04

Values are expressed as mean ± SD. Significant difference between sarcopenic and non-sarcopenic groups: * *p* < 0.05, ** *p* < 0.01, and *** *p* < 0.001. MVIC: maximal voluntary isometric contraction, RMVIC: relative maximal voluntary isometric contraction, TTV: total thigh volume, TFV: thigh fat volume, TMV: thigh muscle volume, TSFV: thigh subcutaneous fat volume, IMAT: thigh intermuscular adipose tissue, 95% CI: 95% confidence interval.

**Table 4:** Comparison of muscle quality and biomarkers between sarcopenic and non-sarcopenic groups.

	Variable	Odds Ratio	*p*-Value	95% CI	
				Lower	Upper
Muscle quality	MVIC (N·m)	0.968	0.004	0.947	0.990
	RMVIC (N·m·kg^−1^)	0.989	0.032	0.979	0.999
	TTV (cm^2^)	0.974	0.077	0.947	1.003
	TFV (cm^2^)	1.039	0.063	0.998	1.081
	TMV (cm^2^)	0.836	0.000	0.756	0.923
	TSFV (cm^2^)	1.027	0.212	0.985	1.072
	IMAT (cm^2^)	1.138	0.037	1.008	1.284
Muscle growth factor	GDF-15 (pg·mL^−1^)	1.001	0.303	0.999	1.002
	GDF-8 (pg·mL^−1^)	1.000	0.783	0.999	1.001
	Activin A (pg·mL^−1^)	1.003	0.245	0.998	1.009
	Follistatin (pg·mL^−1^)	1.000	0.458	0.999	1.001

MVIC: maximal voluntary isometric contraction, RMVIC: relative maximal voluntary isometric contraction, TTV: total thigh volume, TFV: thigh fat volume, TMV: thigh muscle volume, TSFV: thigh subcutaneous fat volume, IMAT: thigh intermuscular adipose tissue, 95% CI: 95% confidence interval.
